# Novel dominant K_ATP_ channel mutations in infants with congenital hyperinsulinism: Validation by in vitro expression studies and in vivo carrier phenotyping

**DOI:** 10.1002/ajmg.a.61335

**Published:** 2019-08-28

**Authors:** Kara E. Boodhansingh, Balamurugan Kandasamy, Lauren Mitteer, Stephanie Givler, Diva D. De Leon, Show‐Ling Shyng, Arupa Ganguly, Charles A. Stanley

**Affiliations:** ^1^ Division of Endocrinology and Diabetes The Children's Hospital of Philadelphia Philadelphia Pennsylvania; ^2^ Department of Pediatrics Perelman School of Medicine at the University of Pennsylvania Philadelphia Pennsylvania; ^3^ Department of Biochemistry and Molecular Biology Oregon Health & Science University Portland Oregon; ^4^ Department of Genetics The Perelman School of Medicine at the University of Pennsylvania Philadelphia Pennsylvania

**Keywords:** diazoxide, genetics, hypoglycemia, pancreatectomy, pancreatic beta‐cells

## Abstract

Inactivating mutations in the genes encoding the two subunits of the pancreatic beta‐cell K_ATP_ channel, *ABCC8* and *KCNJ11*, are the most common finding in children with congenital hyperinsulinism (HI). Interpreting novel missense variants in these genes is problematic, because they can be either dominant or recessive mutations, benign polymorphisms, or diabetes mutations. This report describes six novel missense variants in *ABCC8* and *KCNJ11* that were identified in 11 probands with congenital HI. One of the three *ABCC8* mutations (p.Ala1458Thr) and all three *KCNJ11* mutations were associated with responsiveness to diazoxide. Sixteen family members carried the *ABCC8* or *KCNJ11* mutations; only two had hypoglycemia detected at birth and four others reported symptoms of hypoglycemia. Phenotype testing of seven adult mutation carriers revealed abnormal protein‐induced hypoglycemia in all; fasting hypoketotic hypoglycemia was demonstrated in four of the seven. All of six mutations were confirmed to cause dominant pathogenic defects based on in vitro expression studies in COSm6 cells demonstrating normal trafficking, but reduced responses to MgADP and diazoxide. These results indicate a combination of in vitro and in vivo phenotype tests can be used to differentiate dominant from recessive K_ATP_ channel HI mutations and personalize management of children with congenital HI.

## 1 INTRODUCTION

Congenital hyperinsulinism (HI) due to genetic disorders of pancreatic beta‐cell insulin secretion is the most common and most severe cause of persistent hypoglycemia in infants and children. Rapid diagnosis and treatment are necessary to prevent seizures and permanent brain damage from hypoglycemia. Genetic loci associated with HI include *ABCC8*, *KCNJ11*, *GLUD1*, *GCK*, *HADH‐1*, *SLC16A1*, *UCP2*, *HNF4A*, *HNF1A*, *KDM6A*, *KMT2D*, *CACNAD1*, *FOXA2*, and *KCNQ1* (Aguilar‐Bryan et al., 1995; Clayton et al., 2001; Dusatkova et al., 2011; Ferrara et al., 2017; Flanagan et al., 2010; Flanagan et al., 2017; Giri et al., 2017; Glaser et al., 1998; Gonzalez‐Barroso et al., 2008; Heslegrave et al., 2012; Li et al., 2010; Molven et al., 2004; Nestorowicz et al., 1996; Nestorowicz et al., 1997; Otonkoski et al., 2003; Pearson et al., 2007; Pingul, Hughes, Wu, Stanley, & Gruppuso, 2011; Stanescu, Hughes, Kaplan, Stanley, & De Leon, 2012; Stanley et al., 1998; Thomas et al., 1995; Torekov et al., 2014; Tung, Boodhansingh, Stanley, & De Leon, 2018; Yap et al., 2019). The most common defects are loss of function mutations in *ABCC8* and *KCNJ11* on chromosome 11p that encode SUR1 and Kir6.2, the two subunits of the beta‐cell K_ATP_ potassium channel. In a large series of cases seen at The Children's Hospital of Philadelphia, over 90% of children that were unresponsive to medical therapy with diazoxide, a K_ATP_ channel agonist, were found to have inactivating K_ATP_ channel mutations (Snider et al., 2013). Inactivating K_ATP_ channel mutations were also the most common defects found among diazoxide‐responsive HI cases.

K_ATP_ channel HI mutations can act in either recessive or dominant fashion, depending on the specific defect. Homozygous or compound heterozygous recessive K_ATP_ channel mutations cause diffuse HI that is unresponsive to medical treatment with diazoxide and, therefore, many of these patients require near‐total pancreatectomy to control hypoglycemia. Recessive K_ATP_ channel mutations can also cause focal diazoxide‐unresponsive HI when a paternally‐transmitted mutation becomes isodisomic due to somatic loss of the maternal 11p imprinted region (11pUPD; de Lonlay et al., 1997). Dominant K_ATP_ channel HI mutations are less common than recessive mutations; however, they account for all K_ATP_‐HI cases with diazoxide‐responsive HI and for approximately one‐third of cases with diazoxide‐unresponsive diffuse K_ATP_‐HI (Macmullen et al., 2011; Pinney et al., 2008; Snider et al., 2013). Dominant K_ATP_ channel mutations may be difficult to distinguish from recessive mutations based on family history, because clinical manifestations in carriers of dominant mutations may fail to be recognized (Macmullen et al., 2011; Pinney et al., 2008).

Genetic diagnosis has become an essential tool for clinical management of children with congenital HI, particularly since identification of a monoallelic paternally‐transmitted recessive K_ATP_ channel mutation can accurately diagnose the presence of a focal HI lesion (Snider et al., 2013). In these cases, ^18^F‐DOPA PET/CT scan can be used to localize the lesion and guide curative surgical excision (Laje et al., 2013; Otonkoski et al., 2006). Unfortunately, one‐third to one‐half of K_ATP_ channel mutations detected by genetic testing are novel variants that are interpreted as variants of unknown significance. This is particularly a problem with novel missense K_ATP_ channel variants, since these can potentially be benign polymorphisms, recessive inactivating mutations, dominant inactivating mutations, or even dominant diabetes‐causing mutations (Babenko et al., 2006; Gloyn et al., 2004; Snider et al., 2013). In silico prediction software is not able to accurately predict whether novel missense K_ATP_ channel variants are pathogenic and cannot identify the mode of inheritance (Adzhubei et al., 2010; Sim et al., 2012). The ability to categorize a novel mutation as dominant or recessive is also important for counseling families about recurrence risk and for identifying other family members at risk of hypoglycemia. Thus, characterization of novel variants and their associated phenotypes is essential for improving the interpretation of genetic tests and for accurate diagnosis of affected children. For this purpose, the present report adds six novel dominant K_ATP_ channel mutations to the 62 mutations currently known. These novel mutations were confirmed to be dominant based both on in vitro expression studies and by in vivo phenotype tests of mutation carrier parents.

## 2 METHODS

### 2.1 Consent

Written informed consent was provided by all subjects or their parents. This study was approved by the Children's Hospital of Philadelphia Institutional Review Board (IRB 05‐004037, IRB 07‐005772, IRB 09‐007046).

### 2.2 Probands

Probands were identified during inpatient evaluations in the Congenital Hyperinsulinism Center at The Children's Hospital of Philadelphia between 2006 and 2018. The diagnoses of HI was based on previously described criteria: fasting hypoglycemia accompanied by inadequate suppression of plasma insulin, inappropriate suppression of plasma free fatty acid and β‐hydroxybutyrate concentrations, and inappropriate glycemic response to glucagon at the time of hypoglycemia (Finegold, Stanley, & Baker, 1980; Stanley & Baker, 1976). Patients were defined as being responsive to diazoxide if HI could be completely controlled by treatment with diazoxide at doses ≤15 mg/kg/day, as demonstrated by maintaining plasma glucose concentrations ≥70 mg/dl for 12–18 hr of fasting and/or by developing appropriate fasting hyperketonemia (β‐hydroxybutyrate >2 mmol/l with concurrent plasma glucose <50 mg/dl).

### 2.3 Family members of probands

Family members of probands were identified by parent of origin testing for genetic mutations found in the probands. Family members completed a thorough review of their hypoglycemic history, awareness of symptoms, and any treatment.

### 2.4 Mutation screening

Mutation screening was performed in commercial laboratories for all probands and parents included in this study. Additional mutation analysis for other family members was performed on a research basis. Genomic DNA was isolated from peripheral blood (5 PRIME, Gaithersburg, MD) or from saliva (Oragene DNA self‐collection kit; DNA Genotek, Kanata, Ontario, Canada). Coding sequences and intron/exon splice junctions were amplified and directly sequenced on an ABI 3730 capillary DNA analyzer (Applied Biosystems, Carlsbad, CA). The nucleotides of ABCC8 and corresponding SUR1 amino acids were numbered according to the sequence reported by Nestorowicz et al. (1996) that includes the alternatively spliced exon 17 sequence (NCBI accession no. L78224). The functional consequences of novel, missense mutations were predicted with bioinformatics software SIFT (Sim et al., 2012) and PolyPhen2 (Adzhubei et al., 2010). Genetic variants were searched against the gnomAD Browser (v2.1; Lek et al., 2016).

### 2.5 Phenotype studies

Fasting test: Subjects were fasted for 20–24 hr during which time blood samples were taken at regular intervals to measure plasma glucose, β‐hydroxybutyrate, and insulin. Fasting tests were terminated after 20–24 hr or when plasma glucose fell to <50 mg/dl (2.8 mmol/l).

Oral protein tolerance test (oPTT): Subjects consumed a protein drink containing 1.5 g/kg of protein powder (maximum 60 g; Resource Instant Beneprotein Powder, Nestlé Health Science) mixed with water and sugar‐free flavoring (Sugar‐free Crystal Light™, Kraft Foods, Inc.) after at least 3 hr of fasting. Plasma glucose concentrations were measured at baseline and every 30 min for 3 hr. Abnormal responses were interpreted as either a decrease of plasma glucose greater than 10 mg/dl (0.56 mmol/l) or a drop in plasma glucose to below 70 mg/dl (3.9 mmol/l; Fourtner, Stanley, & Kelly, 2006; Hsu et al., 2001).

Oral glucose tolerance test (oGTT): Subjects consumed a glucose drink containing 1.75 g/kg of glucose (maximum 75 g) after at least 3 hr of fasting (NERL™ Trutol™, ThermoFisher Scientific™, East Providence, RI). Blood samples were taken at baseline and 30 min for 3–4 hr. Results were compared to published norms for adults (American Diabetes, 2010). Abnormal response to glucose was interpreted as a peak glucose >150 mg/dl (8.3 mmol/l).

### 2.6 Mutation expression studies

Plasmids and transfection: Human *ABCC8* cDNA encoding SUR1 (NM_000352.4) was cloned into pCMV6b and human *KCNJ11* cDNA encoding Kir6.2 (NM_000525.3) was cloned into pCDNA3. Point mutations were introduced using site‐directed mutagenesis PCR according to the manufacturer's instructions (QuikChange, Agilent, Santa Clara, CA). SUR1 cDNA and Kir6.2 cDNA were co‐transfected into COSm6 (kidney cell line derived from African green monkey) at 70–80% confluency using the FuGENE® 6 transfection reagent (Promega, Madison, WI).

Immunoblotting: COSm6 cells grown in 6‐well culture plates were transfected with 0.6 μg human SUR1 and 0.4 μg human Kir6.2 cDNA (~1:1 M ratio) per well using FuGENE® 6 (Roche Applied Science, Penzburg, Germany). Cells were lysed in 20 mM Hepes, pH 7.0/5 mM EDTA/150 mM NaCl/1% Nonidet P‐40 with cOmplete™ protease inhibitors (Roche Applied Science, Penzburg, Germany) 48–72 hr post‐transfection. Proteins were separated by SDS/PAGE (8%), transferred to nitrocellulose, analyzed by a rabbit anti‐SUR1 antibody recognizing the C‐terminal 13 amino acids of SUR1 (Chen et al., 2013) followed by horseradish peroxidase (HRP)‐conjugated anti‐rabbit secondary antibodies (GE Healthcare, Chicago, Illinois), and visualized by enhanced chemiluminescence (Super Signal West Femto; Pierce, ThermoFisher Scientific, Waltham, MA).

^86^Rb^+^ efflux assay: Response of channels to metabolic inhibition was assessed by ^86^Rb^+^ efflux assays. COSm6 cells transfected with human SUR1 and Kir6.2 cDNA were incubated overnight in medium containing ^86^RbCl (1 μCi/mL). Before the assay, cells were washed with Krebs‐Ringer solution and incubated with metabolic inhibitors (1 mM 2‐deoxyglucose and 2.5 μg/mL oligomycin) in the presence of ^86^RbCl for 30 min. Cells were then washed with Krebs–Ringer to remove ^86^Rb^+^ in the medium. ^86^Rb^+^ efflux was monitored over a 40‐min cumulative time period by adding and collecting fresh Krebs–Ringer containing metabolic inhibitors repeatedly at 2.5, 5, 7.5, 15, 25, and 40 min. At the end of the 40 min, cells were lysed with 1% SDS. ^86^Rb^+^ in collected solutions and the cell lysate was counted. The percentage efflux at each time point was calculated as the cumulative counts in the solution(s) divided by the total counts from all solutions plus the cell lysate. To calculate the K_ATP_ channel mediated efflux rate constant, efflux data were fitted to the following equation: efflux = 1‐e^((−*k1t*) + (−*k2t*))^; where *k1* represents the rate constant calculated from untransfected cells and *k2* is the rate constant for the K_ATP_ channel mediated efflux (Cooper, Sala‐Rabanal, Lee, & Nichols, 2015). Because there was a time‐dependent divergence of efflux from the monoexponential fit (due to the effect of metabolic inhibition on background efflux), only data from the first three time points (2.5, 5, 7.5 min) were used for fitting.

Patch‐clamp recordings: Functional properties of channels were studied using the inside‐out patch‐clamp recording technique (Taschenberger et al., 2002). Briefly, COSm6 cells transfected with WT or mutant human channel cDNAs and the green fluorescent protein cDNA (for identification of transfected cells) were recorded 36–72 hr post‐transfection at ‐50 mV. Micropipettes pulled from non‐heparinized Kimble glass (Fisher Scientific, Hampton, NH) had resistance ~1.5–2.0 Ω. The bath (intracellular) and pipette (extracellular) solution (K‐INT) contained 140 mM KCl, 10 mM K‐HEPES, 1 mM K‐EGTA, pH 7.3. ATP and ADP were added as potassium salts and free [Mg^2+^] was at ~1 mM in all nucleotide‐containing solutions. Data were analyzed using pCLAMP (Axon Instrument, Molecular Devices, San Jose, CA). Currents in MgADP or diazoxide were calculated as % of currents observed in K‐INT solution.

## 3 RESULTS

### 3.1 Illustrative case (Family 1)

**Figure 1:**
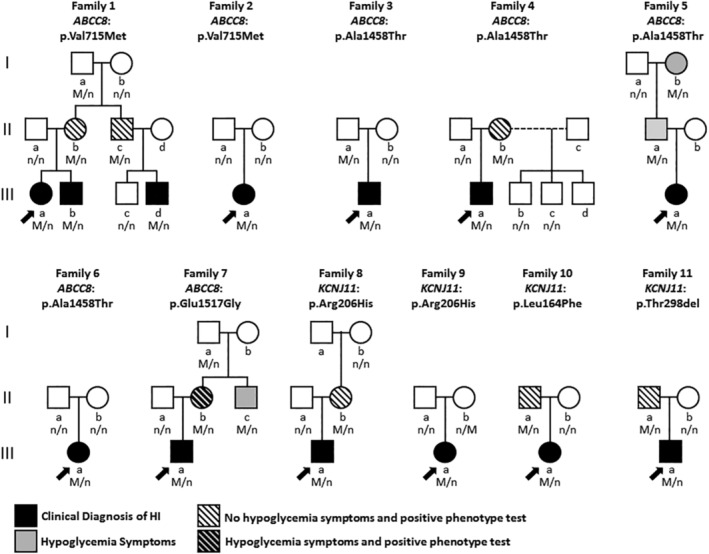
Pedigrees of 11 families with dominant K_ATP_ channel mutations. The pedigrees are listed in ascending order of SUR1 codons followed by Kir6.2 codons. Arrows indicate probands. Black shapes, hypoglycemia diagnosed; gray shapes, symptoms of hypoglycemia reported without clinical confirmation; white and black diagonal lines, hypoglycemia identified on phenotype test without prior knowledge of symptoms; gray and black diagonal lines, hypoglycemia identified on phenotype test and prior knowledge of symptoms. n/M, mutation positive; n/n, mutation negative

The proband in Family 1 (Figure 1 and Table 1, 1‐III‐a) was a female born at 41 weeks gestation (birth weight 3.77 kg, *Z*‐score + 0.57) following an uncomplicated pregnancy, labor, and delivery. She appeared to be growing and developing normally until 9 months of age when her parents first noted an episode of weakness, drooling, stupor, and pallor. This was interpreted as possible apnea and she was taken to an emergency department where an EKG and EEG were found to be normal. One month later at day care, she had two brief episodes of cyanotic apnea that lasted less than a minute; a plasma glucose test was not done. Subsequently, her parents witnessed an episode of shaking during sleep, after which she awakened in a stupor. She was again taken to an emergency department where the plasma glucose was 29 mg/dl (1.6 mmol/l). Hypoglycemia was confirmed by a 6‐hr fasting test, which was followed by an inappropriately large glycemic response (delta 73 mg/dl) to a pharmacologic dose of glucagon. A diagnosis of HI was made and she was transferred to The Children's Hospital of Philadelphia. Treatment with diazoxide at a high dose of 20 mg/kg/day failed to control the hypoglycemia: the plasma glucose fell to 50 mg/dl (2.8 mmol/l) after only 5 hr of fasting with suppressed plasma beta‐hydroxybutyrate concentration (0.82 mmol/l) and an inappropriately large glycemic response to glucagon (delta 40 mg/dl). Diazoxide treatment was then discontinued. After an ^18^F‐DOPA PET/CT scan failed to detect a focal lesion, the child was begun on treatment with octreotide, 11 mcg/kg/day, together with overnight continuous intragastric infusion of dextrose at 5 mg/kg/min. On this regimen, she was able to fast for 3–6 hr and discharged home. At 22 months of age, lanreotide, a long‐acting somatostatin analog, was begun and overnight tube feedings were discontinued. At 4 years of age, she maintains good glycemic control on monthly injections of lanreotide.

**Table 1:** Six novel dominant K_ATP_ channel mutations identified in 11 probands with hyperinsulinism

Gene	Nucleotide change	Amino acid substitution	SIFT	PP2	Allele frequency^a^	Reference (PMID)
*ABCC8*	c.2143g>a	p.Val715Met	D	PrD	–	–
*ABCC8*	c.4372g>a	p.Ala1458Thr	D	PrD	–	12364426, 12627323, 21536946, 27538677
*ABCC8*	c.4550a>g	p.Glu1517Gly	D	B	–	–
*KCNJ11*	c.617g>a	p.Arg206His	D	PrD	–	–
*KCNJ11*	c.490c>t	p.Leu164Phe	D	PrD	–	–
*KCNJ11*	c.892_894del	p.Thr298del	–	–	–	–

a Based on Genome Aggregation Database (gnomAD) v2.1, which includes data from 141,456 individuals.

**Figure 2:**
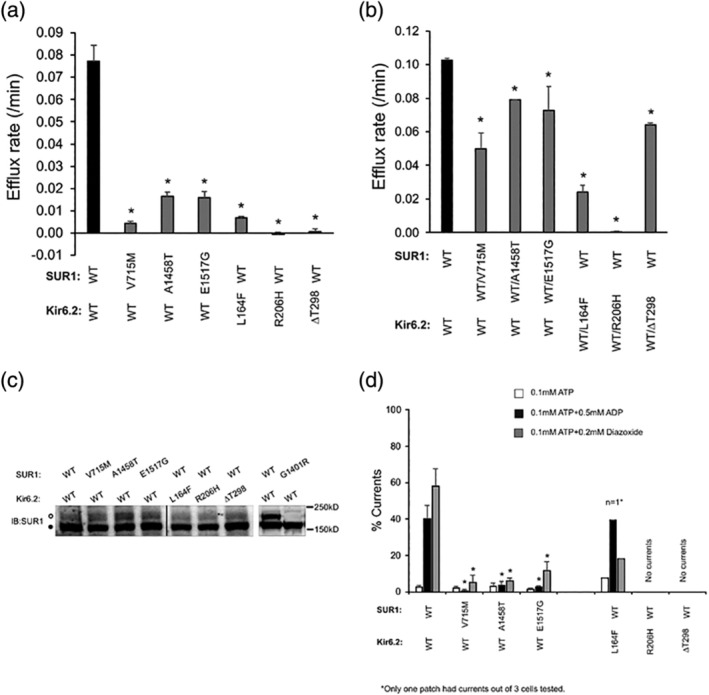
Functional characterization of mutant channels. (a) ^86^Rb^+^ efflux rate calculated using the equation described in the methods. Data represent mean ± *SEM* of three experiments. *p < .01, comparison by two‐tailed, unpaired Student's *t* test. (b) Same as (a) except mutant protein was expressed in the simulated heterozygous states (WT:mutant at 1:1 cDNA ratio). (c) Western blot analysis of SUR1 in COSm6 cells transiently transfected with the various combinations of WT or mutant SUR1 or Kir6.2 as indicated above the blot. The steady state levels of both lower and upper bands of SUR1 of mutant channels were comparable to SUR1 of WT channels. The vertical line separates two parts of the same blot. (d) Inside‐out patch‐clamp recording of WT and mutant channels expressed in COSm6 cells. Mg‐nucleotides response was monitored by exposing recombinant WT or mutant channels to solutions containing 0.1 mM ATP, 0.1 mM ATP + 0.5 mM ADP, or 0.1 mM ATP + 0.2 mM diazoxide, all with 1 mM free Mg^2+^. Baseline currents obtained by exposing the channels to 1 mM ATP were subtracted. Currents in the various solutions were expressed as % of the currents seen in K‐INT (140 mM KCl, 10 mM K‐HEPES,1 mM K‐EGTA. pH 7.2). Currents were measured at −50 mV in symmetrical K‐INT solution, and inward currents are shown as upward deflections. Each bar represents mean ± *SEM* of 4–10 patches. *p < .05, ^**^ *p* < .01, comparison between the mutant and WT, by two‐tailed, unpaired Student's *t* test

Mutation analysis of genes associated with congenital HI in a commercial laboratory found a maternally‐transmitted novel missense variant in *ABCC8* (p.Val715Met; Table 2) that was interpreted as a “variant of unknown significance.” The mother denied having symptoms of hypoglycemia; however, phenotype testing demonstrated that she was affected (shown in Table 3): a 24‐hr fasting test revealed hypoketotic hypoglycemia; an oPTT provoked an abnormal drop in plasma glucose to 65 mg/dl (3.6 mmol/l; delta 27 mg/dl or 1.5 mmol/l). The mother's oGTT showed mild glucose intolerance; the mother also had gestational diabetes during the pregnancy, consistent with previous reports of gestational diabetes and impaired glucose‐stimulated insulin secretion in patients with K_ATP_ channel inactivating mutations (Grimberg et al., 2001; Huopio et al., 2002). These clinical tests indicated that *ABCC8* (p.Val715Met) was a dominant, disease‐causing mutation which was subsequently confirmed by in vitro expression studies (see Figure 2).

**Table 2:** Molecular and clinical characteristics in 11 probands with novel dominant K_ATP_ channel mutations

Patient ID	Mutation	Parent of origin	Birth weight (*Z*‐score)	Age of presentation	Diazoxide response	Surgery	Latest known management
1‐III‐a	*ABCC8*: p.Val715Met	Maternal	3.8 kg (0.57)	9 months with seizure (diagnosed at 10 months)	No	No	Lanreotide
2‐III‐a	*ABCC8*: p.Val715Met	Not maternal	5.2 kg (3.17)	DOL1	No	No	Lanreotide
3‐III‐a	*ABCC8*: p.Ala1458Thr	Paternal	2.9 kg (−0.74)	DOL 1 (diagnosed at 6 months)	No^a^	No (biopsies)	Diazoxide
4‐III‐a	*ABCC8*: p.Ala1458Thr	Maternal	4.8 kg (3.43)	DOL1	Yes	No	Diazoxide
5‐III‐a	*ABCC8*: p.Ala1458Thr	Paternal	3.9 kg (0.53)	DOL1 (diagnosed at 15 months)	Yes	No	Diazoxide until age 7; currently diabetic on metformin at 31 years
6‐III‐a	*ABCC8*: p.Ala1458Thr	De novo	4.0 kg (1.43)	DOL 1 (diagnosed at 16 months)	Yes	No	Diazoxide
7‐III‐a	*ABCC8*: p.Glu1517Gly	Maternal	4.7 kg (2.73)	DOL1	No	No	Diazoxide+octreotide
8‐III‐a	*KCNJ11*: p.Arg206His	Maternal	4.6 kg (5.32)	DOL1	Yes	No	Diazoxide
9‐III‐a	*KCNJ11*: p.Arg206His	Unknown	3.7 kg (0.92)	DOL1	Yes	No	Lanreotide
10‐III‐a	*KCNJ11*: p.Leu164Phe	Paternal	2.4 kg (4.52)	DOL1	Yes	No	Diazoxide
11‐III‐a	*KCNJ11*: p.Thr298del	Paternal	5.2 kg (5.19)	DOL1	Yes	No	Diazoxide

a Patient 3 was partially responsive to diazoxide and pancreatic biopsies showed diffuse disease; parents refused surgery.

**Table 3:** Clinical characteristics and phenotype results in carrier parents

					Fast			oPTT			oGTT		
						Glucose, mg/dl	BOHB, mmol/l	Glucose, mg/dl			Glucose, mg/dl		
Patient ID	Relationship to Proband	Age, years	BMI	Hypoglycemia symptoms	Duration, hr	Final	Final	Basal	Nadir	Delta	Basal	Peak	Nadir
1‐II‐b	Mother	39	19.9	No	19.83	50	0.5	92	65	27	62	153	30
1‐II‐c	Maternal uncle	32	29.1	No	–	–	–	103	66	37	–	–	–
4‐II‐b	Mother	38	37.5	No	22.75	61	0.1	93	68	25	–	–	–
7‐II‐b	Mother	25	22.5	Yes	23.5	55	0.3	90	74	16	80	144	56
8‐II‐b	Mother	30	22.6	No	22.5	73	0.7	108	70	38	–	–	–
10‐II‐a	Father	27	36.9	No	23.5	74	0.4	114	67	47	84	197	57
11‐II‐a	Father	29	34.2	No	24	63	0.3	99	77	22	–	–	–

The proband's younger brother (1‐III‐b) was born at 39 weeks gestation (birth weight 3.9 kg, *Z*‐score + 0.96). Because of the history of HI in his older sister, plasma glucose was measured at birth and found to be 37 mg/dl (2.0 mmol/l); continuous intravenous dextrose infusion was required to control hypoglycemia. During his first week of life, a diagnostic fasting test revealed hypoglycemia following only 3.5 hr of fasting (plasma glucose 35 mg/dl, 2.0 mmol/l) with suppressed beta‐hydroxybutyrate and an inappropriately large glycemic response to glucagon (delta 82 mg/dl), confirming HI. Targeted genetic testing revealed that 1‐III‐b also carried the *ABCC8* p.Val715Met mutation and further confirmed that the mutation acted in dominant fashion. After transfer to The Children's Hospital of Philadelphia, a trial of diazoxide at 13 mg/kg/day failed to control hypoglycemia, indicating that he also had diazoxide‐unresponsive HI. His hypoglycemia was successfully managed with intragastric dextrose at 9 mg/kg/min and at 6 months of age octreotide therapy was added. At 10 months of age, treatment was switched to monthly injections of lanreotide in combination with overnight continuous infusion of dextrose. His hypoglycemia continues to be controlled on this regimen at 2 years of age.

Cascade genetic testing of other family members showed that both the proband's maternal uncle (1‐II‐c), and his 2‐year‐old son (1‐III‐d) also carried the ABCC8 p.Val715Met mutation. While the maternal uncle denied any symptoms of hypoglycemia, his oPTT revealed an abnormal hypoglycemic response, reconfirming that *ABCC8* p.Val715Met was a pathogenic dominant mutation (see Table 3). Review of his son's birth history revealed that 1‐III‐d had low plasma glucose detected at birth requiring treatment with continuous intravenous dextrose infusions; however, his plasma glucose was thought to normalize and he was discharged home at 1 week of age. After 1‐III‐d was found to be heterozygous for the *ABCC8* p.Val715Met mutation, his parents tested plasma glucose levels at home and he was found to have abnormal low overnight fasting values of 50–60 mg/dl (2.8–3.3 mmol/l). Evaluation at The Children's Hospital of Philadelphia confirmed that 1‐III‐d had congenital HI, but appeared to be only mildly affected and did not require medical therapy (fasting tolerance of 12–15 hr).

### 3.2 Clinical features of novel dominant K_ATP_ channel HI mutations

We analyzed six novel K_ATP_ channel variants as possible pathogenic dominant HI mutations by clinical phenotyping of carrier parents and by in vitro expression studies. Five of these mutations were novel and one had previously been reported both as a dominant and a recessive defect (*ABCC8*: p.Ala1458Thr; Huopio et al., 2002; Macmullen et al., 2011; Ovsyannikova et al., 2016; Reimann et al., 2003). As shown in Table 1, three of the novel mutations were in *ABCC8* and three in *KCNJ11*. All were single amino acid variants: five were missense substitutions while the sixth was a deletion of a single amino acid. All five missense substitutions were predicted to be damaging by SIFT and/or PolyPhen. All six mutations were very rare since none were listed in population frequency databases (gnomAD). As shown in Figure 1, these six novel mutations were identified in 13 children with clinically‐diagnosed HI from 11 families. Six of these children inherited their mutation from their mother and five from their father; mutations in the remaining two cases arose de novo.

As shown in Table 2, seven of the 11 probands had birth weights that were large for gestational age (a common feature of congenital HI due to the growth‐promoting effects of insulin in utero) and four had birth weights that were appropriate for gestational age. Twelve of the 13 affected children presented with hypoglycemia on the first day of life. However, in three of these, the diagnosis of HI was not made until much later (6, 15, and 16 months, respectively). One proband did not present with hypoglycemia symptoms (seizure) until 9 months of age. Seven of the 11 probands were responsive to medical therapy with diazoxide, four were not responsive. Proband 3‐III‐a was not fully responsive to diazoxide. Biopsies of her pancreas showed diffuse disease; her parents declined further surgery and she was ultimately managed on diazoxide. The remaining three children who were diazoxide unresponsive were managed with octreotide or lanreotide plus tube feedings and did not have pancreatectomies.

Among the 11 families shown in Figure 1, 16 family members were found to carry the same novel K_ATP_ channel mutation as the proband. As described in the illustrative case, two of these family members had been separately diagnosed with HI in the perinatal period (1‐III‐b and 1‐III‐d). Of the remaining 14, only four individuals from two families gave a history of hypoglycemia (4/14, 29%; 5‐II‐a, 5‐I‐b, 7‐II‐b, 7‐II‐c), while the remaining carriers (71%) all denied symptoms. In Family 5, the proband's father and paternal grandmother had self‐reported symptoms of hypoglycemia which had not been confirmed clinically; both carried the mutation identified in the proband (*ABCC8*: p.Ala1458Thr). In Family 7, the proband's mother reported undiagnosed symptoms of hypoglycemia in herself and her brother; both were found to carry the mutation identified in the proband (*ABCC8*: p.Glu1517Gly).

### 3.3 Phenotype testing of adult carriers

Seven carrier adults from six of the families (1, 4, 7, 8, 10, 11) underwent phenotype testing, including a 24‐hr fasting test, oPTT, and oGTT. Clinical features of these carriers are shown in Table 3. Four were female and three were male. Ages ranged from 25 to 39 years of age and BMI ranged from 22.5 to 37.5 kg/m^2^. Six of the seven tested carriers denied having symptoms of hypoglycemia; 7‐II‐b reported symptoms of hypoglycemia that had not been clinically confirmed.

Four of the six carrier adults who underwent a 24‐hr fast ended with a plasma glucose below 70 mg/dl and suppressed ketones, consistent with HI. All of the seven adult carriers who underwent an oPTT had a fall in plasma glucose levels of greater than 15 mg/dl; four of the seven also dropped their plasma glucose levels below 70 mg/dl (3.9 mmol/l). Based on previous studies in control subjects and patients with K_ATP_‐HI and HI due to *GLUD1* activating mutations, all of the carriers were classified as protein sensitive (Fourtner et al., 2006; Hsu et al., 2001). Huopio and Laakso first described evidence of mild glucose intolerance in K_ATP_‐HI, based on responses to oral glucose in families with the Finnish *ABCC8* founder mutation for dominantly inherited congenital HI (Huopio et al., 2000). However, only mild abnormalities were seen in adult carriers in our series, and, therefore, not all were investigated; of the three carriers who underwent an oGTT, two had mild to moderate glucose intolerance (glucose peak >150 mg/dl) and all three had a glucose nadir <60 mg/dl (3.3 mmol/l).

### 3.4 Locations of novel dominant mutation on the K_ATP_ channel structure

**Figure 3:**
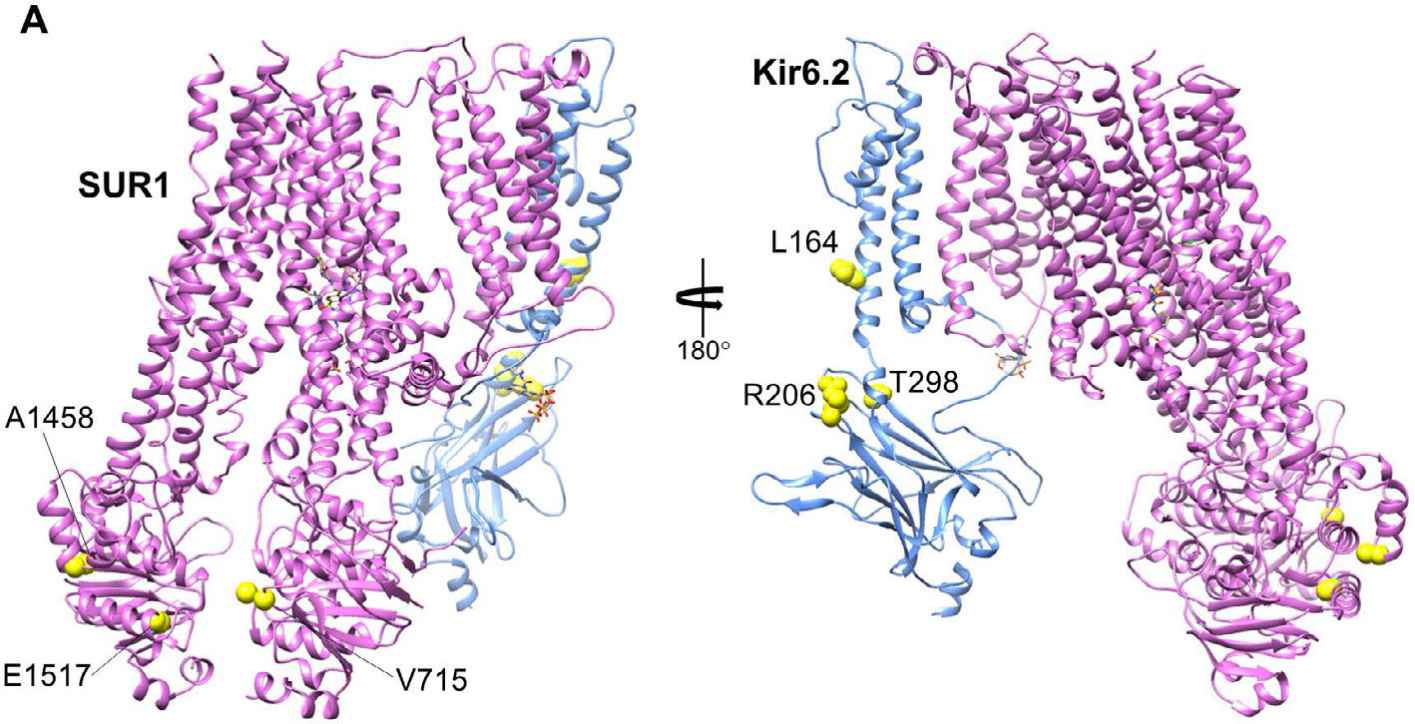
CryoEM K_ATP_ channel structure (PDB:6BAA): Mutation residues are shown in yellow spheres. For clarity, an SUR1 and Kir6.2 dimer in two different side views are shown. SUR1 is colored in purple and Kir6.2 in blue. Glibenclamide and ATP bound to the channel are shown as sticks

Figure 3 shows the locations of the six mutated residues mapped onto the recently solved cryoEM K_ATP_ channel structure (Protein Data Bank [PDB]: 6BAA; Martin, Kandasamy, DiMaio, Yoshioka, & Shyng, 2017; Martin, Yoshioka, et al., 2017). The three mutated SUR1 residues (p.Val715, p.Ala1458, and p.Glu1517) are all in the two nucleotide binding domains (NBD) of SUR1, with p.Val715 in NBD1 and p.Ala1458 and p.Glu1517 in NBD2. Mutations of these residues are predicted to impact the ability of the channel to respond to Mg‐nucleotide and diazoxide stimulation, which requires Mg‐nucleotide binding and/or hydrolysis‐induced NBD dimerization. For residues mutated in Kir6.2, p.Leu164 is situated in the pore‐lining helix near the bundle crossing gate, which is controlled by ATP and phosphatidylinositol 4,5‐bisphosphate (PIP_2_); mutation of this residue has been previously shown to impair channel gating (Haider, Antcliff, Proks, Sansom, & Ashcroft, 2005; Loussouarn, Makhina, Rose, & Nichols, 2000; Phillips, Enkvetchakul, & Nichols, 2003). Residues p.Arg206 and p.Thr298 are both located in the C‐terminal cytoplasmic domain of Kir6.2; the former implicated in both ATP and PIP_2_ sensitivity (Shyng, Cukras, Harwood, & Nichols, 2000) while the latter is close to the G‐loop, which forms the cytoplasmic gate of the channel (Martin, Yoshioka, et al., 2017).

### 3.5 In vitro expression studies of novel dominant K_ATP_ channel HI mutations

#### 3.5.1 Immunoblotting

To assess the molecular effects of these six novel amino acid mutations on the K_ATP_ channel, we first determined the processing efficiency of mutant channels by examining the glycosylation patterns of SUR1 by western blotting (Figure 2c). Complex‐glycosylation of SUR1, which requires successful assembly and trafficking of the channel beyond the medial Golgi and correlates well with channel expression in the membrane, was used to monitor channel maturation and surface expression as described in our previous studies (Macmullen et al., 2011; Pinney et al., 2008; Yan et al., 2007). Lysates from cells expressing wild‐type SUR1 and Kir6.2 or mutant SUR1 and Kir6.2 were analyzed using an anti‐SUR1 antibody (Martin et al., 2016; Yan et al., 2007). All six mutant channels (*ABCC8*: p.Val715Met, p.Ala1458Thr, p.Gly1517Glu; *KCNJ11*: p.Leu164Phe, p.Arg206His, p.Thr298del) showed abundant complex‐glycosylated SUR1 upper band similar to WT channels. These results indicate that the mutations have little impact on channel protein folding or assembly, which is characteristic of dominant K_ATP_ channel mutations identified in congenital HI, to date (Macmullen et al., 2011; Pinney et al., 2008).

#### 3.5.2 ^86^Rb^+^ efflux assay

To assess how the mutations impact the ability of the K_ATP_ channel to open in response to metabolic inhibition, we measured ^86^Rb^+^ efflux in cells expressing WT or mutant channels and pretreated with metabolic inhibitors, as described in the Methods. As shown in Figure 2, among the six mutations, p.Ala1458Thr and p.Glu1517Gly in SUR1 significantly reduced the efflux rate, and p.Val715Met in SUR1 as well as p.Leu164Phe, p.Arg206His, and p.Thr298del in Kir6.2 reduced the efflux rate to nearly 0 after subtracting background efflux observed in non‐transfected cells (see Section 2), confirming a causal role of these mutations in the disease (Figure 2a). Because all six mutations are heterozygous, we also simulated heterozygous expression of the mutants and assessed how the resulting channels responded to metabolic inhibition in ^86^Rb^+^ efflux assays. Figure 2b shows that all mutations caused a significant reduction in the efflux rate, even in simulated heterozygous expression conditions.

#### 3.5.3 Electrophysiology of expressed mutant K_ATP_ channels

Intracellular ATP and ADP are two key physiological regulators that determine the net activity of K_ATP_ channels. ATP and, to a much lesser extent, ADP can inhibit channel opening via Kir6.2 and this inhibition does not require Mg^2+^. On the other hand, ATP and ADP can stimulate channel activity, in a Mg^2+^‐dependent way, by interacting with and causing dimerization of the nucleotide binding domains of SUR1 (Nichols, 2006). Physiological changes in ATP and ADP concentrations as blood glucose levels fluctuate will tip the balance toward ATP inhibition or MgATP/MgADP stimulation to favor channel closure or opening, respectively. Reduced sensitivity to MgATP/MgADP stimulation is one of the most common defects seen in HI‐causing K_ATP_ channel mutations (Nichols et al., 1996; Shyng et al., 1998). We therefore determined whether the three SUR1 and three Kir6.2 mutations in our study alter the MgATP/MgADP sensitivity of the K_ATP_ channel by comparing channel activity in 0.1 mM MgATP alone versus 0.1 mM MgATP plus 0.5 mM MgADP using inside‐out patch‐clamp recordings (see Section 2; Nichols et al., 1996; Zhou et al., 2010). As shown in Figure 2d, all three SUR1 mutations rendered the channel unable to respond to Mg‐nucleotide stimulation, as channels containing these mutations all had much lower activity in 0.1 mM MgATP plus 0.5 mM MgADP compared to WT channels. In addition, all three SUR1 mutations greatly reduced the ability of diazoxide to stimulate channel activity, although some residual stimulation was still observed, especially in the p.Glu1517Gly mutant. In contrast to the robust currents detected in K‐INT solution for the SUR1 mutation containing channels, little to no currents were detected in the Kir6.2 mutation containing channels despite relatively normal expression as indicated by abundant complex‐glycosylated SUR1 (Figure 2c,d). The variability of these studies for *KCNJ11* mutations is due to the single‐cell based nature of the electrophysiology experiment, since using transient transfection, there is a wide range of expression from cell to cell. These results show that the Kir6.2 mutations likely prevent channel opening by impairing ion conduction or PIP_2_ gating. Previous studies of HI‐causing dominant Kir6.2 mutations have uncovered gating defects ranging from no ion conduction (p.Gly156Arg), reduced channel open probability (*P*_*o*_) due to weakened PIP_2_ response (p.Phe55Leu) or spontaneous inactivation of channel activity (p.Arg301Cys/Gly/His; Lin et al., 2008; Lin, MacMullen, Ganguly, Stanley, & Shyng, 2006; Pinney et al., 2008). Given that the Kir6.2 residues mutated are located in structural elements predicted or known to be important for ion conduction or PIP_2_ gating, the lack of channel activity is not surprising. Interestingly, while the p.Arg206His mutation had a very strong dominant negative effect in simulated heterozygous state, the impact of the p.Leu164Phe was less severe and that of the p. Thr298del mutation was even less under the same expression condition. These observations imply that in the tetrameric channels, the presence of even one p.Arg206His Kir6.2 subunit is detrimental to channel activity, while the other mutations may have a more graded effect.

## 4 DISCUSSION

These studies identified six novel single amino acid mutations in *ABCC8* and *KCNJ11* in infants with congenital HI. These six mutations were detected in 13 children from 11 families. The functional consequences of the mutations were evaluated by in vitro expression studies in cultured COSm6 cells and by in vivo phenotype testing of adult carriers. The results demonstrate that these six variants in the SUR1 and Kir6.2 subunits of the beta‐cell K_ATP_ channel can be classified as autosomal dominant HI mutations, in contrast to the more commonly seen recessive missense mutations.

Previous studies have shown that a major difference between recessive vs dominant K_ATP_ channel HI mutations is that recessive mutations result in mutant proteins unable to traffic to the cell surface while dominant mutations yield mutant proteins that retain the ability to traffic (Cartier, Conti, Vandenberg, & Shyng, 2001; Pinney et al., 2008). The subunits of the K_ATP_ channels, SUR1 and Kir6.2, are assembled into hetero‐octameric channels in the endoplasmic reticulum and then must traffic via the Golgi apparatus where the SUR1 acquires mature N‐linked glycosylation before trafficking to the plasma membrane. Recessive mutations of either subunit characterized to date, ranging from nonsense mutations to missense mutations, result in inability of the mutant channels to traffic to the plasma membrane (Cartier et al., 2001; Crane & Aguilar‐Bryan, 2004; Lin et al., 2008; Nestorowicz et al., 1997; Yan et al., 2007). Thus, patients with diffuse hyperinsulinism due to homozygous or compound heterozygous recessive mutations have a lack of K_ATP_ channels and severe hyperinsulinism that is not responsive to treatment with the channel opener, diazoxide. Heterozygous carriers of recessive K_ATP_ mutations appear to be asymptomatic (Grimberg et al., 2001), presumably because the mutant protein is unable to incorporate into the channel complex and is degraded in the ER while the normal allele is able to generate sufficient numbers of functional channels. In contrast, dominant mutations of K_ATP_ subunits characterized to date are missense mutations that produce subunits which can be incorporated into mature octameric channel complexes capable of trafficking to the plasma membrane, but resulting in disruption of normal channel gating activity. Some of these dominant mutations result in a severe impairment of responsiveness to diazoxide, whereas mutations associated with milder impairment of channel gating activity may be diazoxide‐responsive. Recessive K_ATP_ channel mutations can cause focal hyperinsulinism lesions through isodisomy for a paternally derived recessive K_ATP_ channel mutation as a consequence of loss of the maternal 11p region. Focal lesions due to a dominant mutation have not yet been described, but in such a case, resection would not be curative due to the ongoing effect of the mutation in the remaining pancreas. In a child with diazoxide‐unresponsive HI who has a novel monoallelic missense mutation in *ABCC8*, determining whether the mutation is dominant or recessive is of great clinical importance, because if the monoallelic mutation is clearly recessive there is >95% likelihood of a focal lesion that can be cured by F‐DOPA PET scan localization and targeted resection (Snider et al., 2013). In contrast, a monoallelic dominant mutation is diagnostic of diffuse disease. Differentiating between dominant and recessive mutations is also essential for counseling about recurrence risk as well as for identifying other family members at risk for hypoglycemia (as illustrated by the Case Summary of Family 1).

A total of 68 monoallelic mutations in *ABCC8* and *KCNJ11* associated with dominant HI have been reported, including the six described here (see Figure S1 and Table S1a,b in the Supporting Information). These dominant mutations have primarily been single amino acid substitutions, but single amino acid deletions and an insertion of two amino acids have also been reported (Kapoor et al., 2011; Shemer et al., 2012; Thornton et al., 2003). Dominant mutations in *ABCC8* have been associated with both diazoxide‐responsive and diazoxide‐unresponsive disease; in contrast, all of the dominant missense HI mutations in *KCNJ11* reported to date have been diazoxide‐responsive (Shyng, Bushman, Pratt, & Zhou, 2012).

Predicting whether novel missense mutations in *ABCC8* and *KCNJ11* are recessive or dominant is not possible based on mutation locations since mutations that impair channel trafficking have been identified throughout both proteins (Martin, Chen, Devaraneni, & Shyng, 2013; Shyng et al., 2012). (See Figure S1 in the Supporting Information comparing the location of the 68 dominant *ABCC8* and *KCNJ11* HI mutations and those of amino acid missense variants associated with recessive K_ATP_‐HI, diabetes, and benign polymorphisms). A further complication for interpreting missense *ABCC8* variants based on location is that different amino acid substitutions at the same residue may cause opposite phenotypes. In fact, multiple mutations resulting in alternate amino acid substitutions of SUR1 result in different phenotypes (HI or diabetes; Flanagan et al., 2009) as well as different inheritance patterns (recessive or dominant HI mutations; Snider et al., 2013). For example, three different missense changes have been reported at residue 1,458 in *ABCC8*: p.Ala1458Thr, p.Ala1458Pro, and p.Ala1458Val. Expression studies indicate that p.Ala1458Thr (Macmullen et al., 2011) and p.Ala1458Val, (Saint‐Martin et al., 2015) are dominant mutations, while clinical evidence indicates that the third, p.Ala1458Pro, is a recessive (non‐trafficking) mutation since this patient was homozygous and had diffuse HI (Bellanne‐Chantelot et al., 2010). Finally, it is important to note that the number of HI mutations which have been identified in the K_ATP_ channel subunits has not yet reached saturation, since approximately 70% of pathogenic missense HI mutations identified in *ABCC8* and *KCNJ11* continue to be novel variants not previously reported (Snider et al., 2013). Therefore, uncertainty in interpretation of monoallelic novel missense variants of the K_ATP_ subunits continues to be a handicap in applying genetic mutation testing to clinical decision‐making in congenital HI and especially hinders the identification of children with potentially curable focal lesions.

Software programs, such as SIFT (Sim et al., 2012) and PolyPhen2 (Adzhubei et al., 2010), are commonly used to predict whether a novel mutation will be damaging. In the present series of patients, both SIFT and PolyPhen2 correctly predicted that four of the five amino acid substitutions would be damaging (4/5, 80%) but gave conflicting predictions for the fifth (1/5, 20%); neither program could be used for the single amino acid deletion mutation, *KCNJ11*:p.Thr298del. This is similar to the accuracy of these software programs for previously reported dominant and recessive K_ATP_ channel mutations (79% correctly predicted as disease‐associated, 17% had conflicting predictions, and 4% incorrectly predicted to be benign, (Snider et al., 2013). Despite the fact that the accuracy of software programs is high for predicting disease causation for K_ATP_ channel mutations, these programs are not capable of predicting whether a mutation will act recessively or dominantly.

In vitro expression studies of the six novel K_ATP_ channel HI mutations reported here demonstrated features consistent with them acting in a dominant fashion. All were associated with normal trafficking of channels from the Golgi apparatus to the plasma membrane surface in COSm6 cells, whereas recessive mutations severely interfere with normal trafficking (Cartier et al., 2001). All of the six mutations were associated with reduced ^86^Rb^+^ efflux in both homozygous and simulated heterozygous states, confirming that the mutant channels have no or reduced function despite reaching the plasma membrane. All of the three *ABCC8* mutations showed reduced channel currents in response to the channel openers, MgADP and diazoxide.

There are discrepancies concerning the functional consequence and mode of inheritance in some reported cases of missense K_ATP_ channel mutations. Some mutations reported to be dominant or recessive lack adequate supporting evidence, such as clear family history or functional expression data. One example is the *KCNJ11*: p.Gln235Glu mutation reported as a paternally inherited dominant mutation in a child with diazoxide‐unresponsive HI who lacked a dominant family history of hypoglycemia and had not undergone surgery (Sang, Xu, Yan, & Liu, 2012); however, the reported data are insufficient to exclude the possibility that the mutation is recessive and that the patient may have had focal HI. In other cases, there are conflicting in vitro expression studies on whether K_ATP_ channel complexes generated by the mutation interfere with trafficking to the plasma membrane (recessive mutations) or allow normal trafficking with impaired channel activity (dominant). One of the novel dominant mutations found in four families in the present series, *ABCC8*: p.Ala1458Thr, had been previously reported in a child with a single paternally‐transmitted mutation and diffuse HI together with in vitro expression studies using COSm6 cells and hamster SUR1 and rat Kir6.2 constructs showing normal trafficking which suggested a dominant mode of action (MacMullen et al., 2001); this report conflicts with a report of expression studies in *Xenopus* oocytes showing defective surface expression on the plasma membrane indicative of a recessive mutation (Reimann et al., 2003). The present studies confirm a dominant mode of inheritance for p.Ala1458Thr by both in vitro functional studies in COSm6 cells using human SUR1 and Kir6.2 constructs as well as by phenotype testing of an adult carrier that demonstrated protein‐induced hypoglycemia (Family 4).

Functional expression studies using *Xenopus* ooctyes have previously been reported for an *ABCC8*:p.Gly1401Arg variant which concluded that the mutation has little effect on K_ATP_ channel expression levels, implying a dominant mode of action (de Wet et al., 2012); however, in our series of cases, the *ABCC8*:p.Gly1401Arg mutation has been seen only in the context of a recessive mode of action (three cases with focal HI due to a paternally‐transmitted mutation and one case with diffuse HI that had a second recessive mutation, ABCC8: c.2041‐21g>a). As shown in Figure 2c expression of *ABCC8*:p.Gly1401Arg in COSm6 cells showed non‐trafficking K_ATP_ channel complexes, supporting a recessive mode of action and phenotype testing in the carrier mother demonstrated no fasting or protein‐induced hypoglycemia, also consistent with a recessive mutation (data not shown). These examples of discrepant in vitro expression studies of K_ATP_ channel mutations may be due to differences in processing of mutant channels in *Xenopus* oocytes compared to mammalian COSm6 cells. Previous studies have shown that some trafficking‐impaired HI‐causing mutant SUR1 proteins can traffic to the surface in mammalian cells cultured at lower temperature (Yang, Fang, Fromondi, & Chan, 2005). It is also well documented that the most prevalent cystic fibrosis transmembrane conductance regulator (CFTR), p.delPhe508, traffics efficiently to the plasma membrane in *Xenopus* oocytes but not in mammalian cells (Denning et al., 1992; Drumm et al., 1991). Because experiments in *Xenopus* oocytes are carried out at room temperature, rather than 37°C, some non‐trafficking mutant K_ATP_ channels in mammalian cells may be able to traffic to the *Xenopus* oocyte plasma membrane. Based on the present studies, employing a mammalian cell line and using the human constructs for SUR1 and Kir6.2 subunits may be more reliable in evaluating whether novel missense K_ATP_ channel mutations act in dominant or recessive fashion.

It is important to note that family history may not be reliable for identifying a dominant K_ATP_ channel mutation, because mutation carriers may not have been clinically recognized and because adult carriers of dominant HI mutations often deny having symptoms of hypoglycemia (Kukuvitis, Deal, Arbour, & Polychronakos, 1997; Macmullen et al., 2011; Pinney et al., 2008). For example, in the present series of 11 families with novel dominant K_ATP_ channel HI mutations, apart from one infant who was detected at birth due to the family history, only one of the 15 family members who were mutation carriers had documented hypoglycemia (1/15 or 7%); only four had self‐reported symptoms of hypoglycemia (4/15, 27%); and the remaining 10 carriers denied any symptoms of hypoglycemia (10/15, 67%). In contrast, in the current study, all of the six carriers of dominant K_ATP_ channel HI mutations who underwent provocative testing demonstrated evidence of having a hypoglycemia phenotype in response to oral protein (6/6, 100%) and four manifested evidence of hypoketotic hypoglycemia during a 24‐hr fast (4/6, 67%). A high rate of undiagnosed hypoglycemia has been previously noted in reports of dominant congenital HI, including mutations of the K_ATP_ channel, as well as in HI due to dominant activating mutations of glucokinase (Christesen et al., 2002; Dullaart, Hoogenberg, Rouwe, & Stulp, 2004; Glaser et al., 1998; Gloyn et al., 2003; Wabitsch et al., 2007) and glutamate dehydrogenase (Stanley et al., 1998) and in a family with HI associated with the hexokinase‐1 locus (Pinney et al., 2013). Several factors may contribute to the under‐recognition of patients carrying dominant HI mutations, including indications that the hypoglycemia in K_ATP_‐HI may become less severe in adolescents and adults than in young infants (Glaser et al., 1999), failure to diagnose hypoglycemia in children having seizures or other symptoms of hypoglycemia, and possible hypoglycemia unawareness induced by recurrent hypoglycemia (hypoglycemia‐associated autonomic failure [HAAF]; Dagogo‐Jack, Craft, & Cryer, 1993; Segel, Paramore, & Cryer, 2002). The present studies suggest that evidence of HI can be demonstrated in carriers of dominant K_ATP_ channel mutations and that an oral protein tolerance test might be the simplest method for this purpose. Our results also suggest that the penetrance of dominant K_ATP_ channel mutations is near 100%, but that in many carriers hypoglycemia symptoms are not diagnosed.

### 4.1 Limitations

There are several limitations in differentiating between dominant and recessive K_ATP_ channel missense mutations. Pedigrees of patients with novel mutations are often too small to be able to clearly distinguish dominant vs recessive modes of transmission. in vitro mutation expression studies can provide insight into the functional consequences of the amino acid change on channel trafficking and channel activity characteristics of recessive or dominant defects. However, the turnaround time involved in expression studies is generally too long to support clinical decision‐making in a novel case (e.g., defining the possibility of a resectable focal lesion). In addition, there appear to be differences in the reliability of different methods used for expression studies, depending on the type of cell used and the species origin of the constructs for channel subunits; the method employed in the present studies appears to have advantages over studies based on amphibian cells. The results of phenotype testing of mutation carriers can be obtained more quickly than in vitro expression studies to distinguish whether a K_ATP_ channel missense HI mutation is dominant or recessive; as in the present studies, phenotype testing of adult carriers may require only bedside glucose monitoring. The oral protein tolerance test appeared to be more reliable in confirming that carriers were affected than a 24‐hr fasting test or an oral glucose tolerance test. However, since these tests have been carried out only in a limited number of carriers and controls, their accuracy in identifying whether a missense mutation is dominant vs recessive remains uncertain.

## 5 CONCLUSION

In summary, these studies identify six novel K_ATP_ channel inactivating mutations associated with single amino acid changes that cause a dominant form of HI; three in *ABCC8* and three in *KCNJ11*. In silico prediction tools and mutation location were not adequate to define whether these mutations act in dominant vs recessive fashion. However, determination of the functional consequences of the mutations by in vitro expression in a COSm6 mammalian cell line and in vivo phenotype testing of adult carriers confirmed that all six mutations acted in dominant fashion. In vitro expression studies, such as ^86^Rb^+^ efflux, appear to provide the most definitive assessment of pathogenicity; however, results are difficult to obtain quickly enough to inform clinical management. Phenotype studies of mutation carriers, such as the patient's parent, and, in particular, the glucose response to oral protein challenge, may provide a timely method for determining whether a novel K_ATP_ channel missense variant is a dominant HI mutation.

## CONFLICT OF INTEREST

None.

## Supporting information


**Table S1a** Dominant missense mutations reported in *ABCC8* and *KCNJ11*
**Table S1b.** *KCNJ11* mutations reported as dominant but with insufficient evidence

**Figure S1** Supporting information

## Data Availability

The data that support the findings of this study are available from the corresponding author upon reasonable request.
