# Supplementary material for: Novel dominant KATP channel mutations in infants with congenital hyperinsulinism: Validation by in vitro expression studies and in vivo carrier phenotyping
Source: Am J Med Genet A. 2019 Aug 28;179(11):2214–27. doi: 10.1002/ajmg.a.61335 (PMC6852436; doi:10.1002/ajmg.a.61335)
Supplement: Supplementary file 1 — Table S1a Dominant missense mutations reported in ABCC8 and KCNJ11 Table S1b. KCNJ11 mutations reported as dominant but with insufficient evidence [file AJMG-179-2214-s002.docx]

**Supplementary Table 1a. Dominant Missense Mutations Reported in *ABCC8* and *KCNJ11*.**

| ***Gene*** | ***Exon*** | ***Nucleotide*** | ***Codon*** | ***Diazoxide Response*** | ***PMID***  ***(Reporting Dominant)*** | ***dbSNP*** |
| --- | --- | --- | --- | --- | --- | --- |
| *ABCC8* | 2 | c.275 g>a | p.Gly92Asp | - | 26092864 |  |
| *ABCC8* | 3 | c.338 c>t | p.Ala113Val | - | 26092864 |  |
| *ABCC8* | 6 | c.928 g>a | p.Asp310Asn | + | 18596924, 27188453 | rs769569410 |
| *ABCC8* | 7 | c.1063 g>a | p.Ala355Thr | + | 23275527 | rs145136257 |
| *ABCC8* | 7 | c.1108 a>g | p.Arg370Gly | + | 18596924 |  |
| *ABCC8* | 7 | c.1110 g>c | p.Arg370Ser | ? | 18390792 |  |
| *ABCC8* | 8 | c.1222 t>c | p.Ser408Pro | + | 23275527 |  |
| *ABCC8* | 9 | c.1421 a>g | p.Gln474Arg | - | 21536946 |  |
| *ABCC8* | 9 | c.1433 c>a | p.Ala478Asp | - | 21536946 |  |
| *ABCC8* | 10 | c.1531 c>a | p.Leu511Met | - | 24814349 |  |
| *ABCC8* | 10 | c.1562 g>a | p.Arg521Gln | + | 23275527 | rs368114790 |
| *ABCC8* | 16 | c.2143 g>a | p.Val715Met | - | This Report |  |
| *ABCC8* | 16 | c.2144 t>g | p.Val715Gly | - | 21536946 |  |
| *ABCC8* | 16 | c.2144 t>c | p.Val715Ala | - | 21536946 |  |
| *ABCC8* | 16 | c.2147 g>a | p.Gly716Asp | - | 21536946 |  |
| *ABCC8* | 20 | c.2473 g>a | p.Glu825Lys | - | 24814349 |  |
| *ABCC8* | 22 | c.2662 a>c | p.Thr888Pro | - | 21536946 |  |
| *ABCC8* | 22 | c.2669 a>c | p.Lys890Thr | - | 24814349 | rs761862121 |
| *ABCC8* | 22 | c.2672 t>c | p.Leu891Pro | - | 24814349 |  |
| *ABCC8* | 23 | c.2701 a>t | p.Ile901Phe | + | 27188453 |  |
| *ABCC8* | 28 | c.3457 g>a | p.Ala1153Thr | - | 25201519 |  |
| *ABCC8* | 29 | c.3653 g>a | p.Arg1218Lys | + | 25008049 |  |
| *ABCC8* | 31 | c.3787 g>a | p.Ala1263Thr | - | 26092864 |  |
| *ABCC8* | 33 | c.4011 g>c | p.Lys1337Asn | + | 17575084, 23275527 | rs67767715 |
| *ABCC8* | 33 | c.4058 g>a | p.Arg1353His | + | 15356046, 18596924 | rs28936370 |
| *ABCC8* | 33 | c.4060 t>a | p.Tyr1354Asn | + | 27188453 |  |
| *ABCC8* | 33 | c.4100 c>a | p.Ala1367Asp | + | 22308858 |  |
| *ABCC8* | 33 | c.4121 a>g | p.Lys1374Arg | + | 18596924 |  |
| *ABCC8* | 34 | c.4151 g>a | p.Gly1384Glu | - | 21536946 |  |
| *ABCC8* | 34 | c.4153 a>c | p.Lys1385Gln | + | 15087877 |  |
| *ABCC8* | 34 | c.4156 t>c | p.Ser1386Pro | + | 18596924 |  |
| *ABCC8* | 34 | c.4159-4161 delTCC | p.Ser1387del | + | 12941782, 18596924 |  |
| *ABCC8* | 34 | c.4160 c>t | p.Ser1387Phe | - | 21536946 | rs72559718 |
| *ABCC8* | 34 | c.4160 c>a | p.Ser1387Tyr | - | 21536946 |  |
| *ABCC8* | 34 | c.4166 c>a | p.Ser1389Tyr | - | 21536946 |  |
| *ABCC8* | 34 | c.4169 t>g | p.Leu1390Arg | + | 21674179 |  |
| *ABCC8* | 35 | c.4273 a>c | p.Ile1425Leu | - | 26092864 |  |
| *ABCC8* | 35 | c.4291 c>t | p.Leu1431Phe | + | 21674179 |  |
| *ABCC8* | 36 | c.4372 g>a | p.Ala1458Thr | + | 21536946, This Report | rs72559717 |
| *ABCC8* | 36 | c.4373 c>t | p.Ala1458Val | - | 24814349 |  |
| *ABCC8* | 36 | c.4375 c>g | p.Gln1459Glu | + | 21674179 |  |
| *ABCC8* | 36 | c.4377 g>a | p.Gln1459His | - | 21536946 |  |
| *ABCC8* | 37 | c.4433 g>t | p.Gly1478Val | + | 18596924 |  |
| *ABCC8* | 37 | c.4435 g>a | p.Gly1479Arg | + | 18596924, 21674179 | rs72559715 |
| *ABCC8* | 37 | c.4442 a>t | p.Asn1481Ile | - | 24814349 |  |
| *ABCC8* | 37 | c.4454 g>a | p.Gly1485Glu | - | 20573158, 26092864 | rs193922405 |
| *ABCC8* | 37 | c.4518 c>a/g | p.Asp1506Glu | - | 20573158, 26092864, 24814349 |  |
| *ABCC8* | 37 | c.4519 g>a | p.Glu1507Lys | + | 11018078, 18596924 |  |
| *ABCC8* | 37 | c.4522 g>c (reported as g>a) | p.Ala1508Pro | + | 21674179 |  |
| *ABCC8* | 37 | c.4526_4531 dupCGGCTT | p.Ala1510_Ser1511 insSerAla | ? | 22106158 |  |
| *ABCC8* | 37 | c.4535 t>g | p.Ile1512Ser | - | 24814349 |  |
| *ABCC8* | 37 | c.4535 t>c | p.Ile1512Thr | + | 18596924 |  |
| *ABCC8* | 37 | c.4541 t>a | p.Met1514Lys | - | 20573158, 26092864 |  |
| *ABCC8* | 37 | c.4546 a>g | p.Thr1516Ala | + | 27188453 |  |
| *ABCC8* | 38 | c.4549 g>a | p.Glu1517Lys | - | 21536946 |  |
| *ABCC8* | 38 | c.4550 a>g | p.Glu1517Gly | - | This Report |  |
| *ABCC8* | 38 | c.4566 g>t | p.Lys1522Asn | + | 23275527 | rs142272833 |
| *ABCC8* |  | c.4610 c>t | p.Ala1537Val | + | 21674179 | rs745918247 |
| *ABCC8* | 39 | c.4616 g>a | p.Arg1539Gln | + | 18596924, 21674179 |  |
| *KCNJ11* | 1 | c.165 c>a | p.Phe55Leu | + | 18596924 | rs1343400778 |
| *KCNJ11* | 1 | c.185 c>t | p.Thr62Met | + | 23275527 | rs1057518775 |
| *KCNJ11* | 1 | c.466 g>a | p.Gly156Arg | + | 18596924 |  |
| *KCNJ11* | 1 | c.490 c>t | p.Leu164Phe | + | This Report |  |
| *KCNJ11* | 1 | c.612 c>a | p.Asp204Glu | + | 18596924 |  |
| *KCNJ11* | 1 | c.617 g>a | p.Arg206His | + | This Report |  |
| *KCNJ11* | 1 | c.850_852 delATC | p.Ile284del | + | 21674179 |  |
| *KCNJ11* | 1 | c.892_894 delACC | p.Thr298del | + | This Report |  |
| *KCNJ11* | 1 | c.1093 c>t | p.Arg365Cys | + | 23275527 | rs758749160 |

**Supplementary Table 1b. *KCNJ11* mutations reported as dominant but with insufficient evidence.**

| ***Gene*** | ***Exon*** | ***Nucleotide*** | ***Codon*** | ***Diazoxide Response*** | ***PMID***  ***(Reporting Dominant)*** | ***dbSNP*** |
| --- | --- | --- | --- | --- | --- | --- |
| *KCNJ11* | 1 | c.83 c>t | p.Ala28Val | - | 29087246 |  |
| *KCNJ11* | 1 | c.703 c>g | p.Gln235Glu | + | 25008049 |  |

Conventions Used:

Mutations are described using the current Human Genome Variation Society guidelines (den Dunnen et al., 2016). All ABCC8 sequence information is based on GenBank reference sequence NM_00352.2, including the alternate exon 17 (GenBank L78208) which contains an additional amino acid. All KCNJ11 sequence information is based on GenBank reference sequence NM_000525. Population frequency data is from gnomAD Database, accessed March 13, 2019 (Lek et al., 2015).

**References**

den Dunnen, J. T., Dalgleish, R., Maglott, D. R., Hart, R. K., Greenblatt, M. S., McGowan-Jordan, J., . . . Taschner, P. E. (2016). HGVS Recommendations for the Description of Sequence Variants: 2016 Update. *Hum Mutat, 37*(6), 564-569. doi:10.1002/humu.22981

Lek, M., Karczewski, K., Minikel, E., Samocha, K., Banks, E., Fennell, T., . . . MacArthur, D. (2015). Analysis of protein-coding genetic variation in 60,706 humans. *bioRxiv*. doi:10.1101/030338
