# Supplementary figures and images for: Novel dominant KATP channel mutations in infants with congenital hyperinsulinism: Validation by in vitro expression studies and in vivo carrier phenotyping
Source: Am J Med Genet A. 2019 Aug 28;179(11):2214–27. doi: 10.1002/ajmg.a.61335 (PMC6852436; doi:10.1002/ajmg.a.61335)

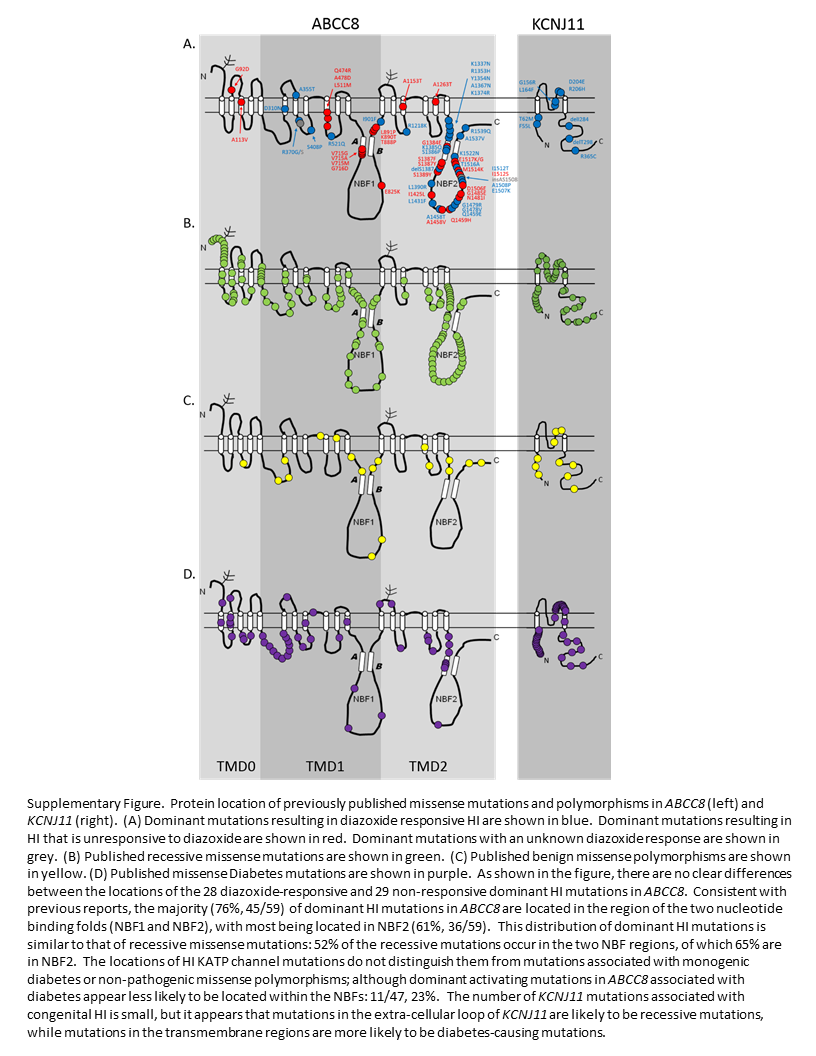

Supplement: Supplementary file 2 — Figure S1 Supporting information [file AJMG-179-2214-s001.TIF]
